# Effect of neck strength training on health-related quality of life in females with chronic neck pain: a randomized controlled 1-year follow-up study

**DOI:** 10.1186/1477-7525-8-48

**Published:** 2010-05-14

**Authors:** Petri K Salo, Arja H Häkkinen, Hannu Kautiainen, Jari J Ylinen

**Affiliations:** 1 Department of Physical and Rehabilitation Medicine, Central Finland Health Care District, Keskussairaalantie 19, FI-40620 Jyväskylä, Finland; 2 Department of Health Sciences, University of Jyväskylä, Jyväskylä, Finland; 3 Unit of Family Practice, Central Hospital of Central Finland, Jyväskylä, Finland; 4 ORTON Foundation, Helsinki, Finland

## Abstract

**Background:**

Chronic neck pain is a common condition associated not only with a decrease in neck muscle strength, but also with decrease in health-related quality of life (HRQoL). While neck strength training has been shown to be effective in improving neck muscle strength and reducing neck pain, HRQoL among patients with neck pain has been reported as an outcome in only two short-term exercise intervention studies. Thus, reports on the influence of a long-term neck strength training intervention on HRQoL among patients with chronic neck pain have been lacking. This study reports the effect of one-year neck strength training on HRQoL in females with chronic neck pain.

**Methods:**

One hundred eighty female office workers, 25 to 53 years of age, with chronic neck pain were randomized to a strength training group (STG, n = 60), endurance training group (ETG, n = 60) or control group (CG, n = 60). The STG performed high-intensity isometric neck strengthening exercises with an elastic band while the ETG performed lighter dynamic neck muscle training. The CG received a single session of guidance on stretching exercises. HRQoL was assessed using the generic 15D questionnaire at baseline and after 12 months. Statistical comparisons among the groups were performed using bootstrap-type analysis of covariance (ANCOVA) with baseline values as covariates. Effect sizes were calculated using the Cohen method for paired samples.

**Results:**

Training led to statistically significant improvement in the 15D total scores for both training groups, whereas no changes occurred for the control group (P = 0.012, between groups). The STG improved significantly in five of 15 dimensions, while the ETG improved significantly in two dimensions. Effect size (and 95% confidence intervals) for the 15D total score was 0.39 (0.13 to 0.72) for the STG, 0.37 (0.08 to 0.67) for the ETG, and -0.06 (-0.25 to 0.15) for the CG.

**Conclusions:**

One year of either strength or endurance training seemed to moderately enhance the HRQoL. Neck and upper body training can be recommended to improve HRQoL of females with neck pain if they are motivated for long-term regular exercise.

**Trial Registration:**

ClinicalTrials.gov NCT01057836

## Background

Neck pain is one of the most common musculoskeletal disorders in Western societies [1-4]. Along with considerable costs for the individual and the society, neck pain is a frequent source of disability causing humane suffering and affecting the well-being of individuals. Just as health is a state of complete physical, mental, and social well-being and not merely the absence of disease or infirmity [5], the outcome measures of an intervention ought to be multidimensional and include the subjective experience of the patient. This can be achieved using a health-related quality of life (HRQoL) measurement tool [6].

Neck pain has been shown to be associated with a decrease in HRQoL in several studies [1,7-12]. While no gold standard exists for assessing HRQoL among patients with neck pain, several different measurement instruments have been used, such as the Short Form-36 Health Survey (SF-36) [13] or subscales of the SF-36, 15 Dimensional HRQoL instrument (15D) [6], EuroQoL Group - 5 dimensional instrument (EQ-5D) [14], and the Healthy Days Measures [15].

Since neck pain is associated with a decrease in neck muscle strength, [16-21] neck strength training has been one means in seeking cure for neck pain. In addition to gaining neck muscle strength, neck strength training has been shown to be effective in reducing neck pain and the disability associated with it [22-24]. In a recent best-evidence synthesis [25] and Cochrane review [26] it was concluded that interventions that involved exercise combined with manual therapy were more effective in treating patients with neck pain than were alternative strategies. Although strength training seems to be an efficient way of treating patients with neck pain, its effect on HRQoL has not been shown. The authors found only two studies where the influence of strength exercises on neck pain was assessed with HRQoL measurements [22,27]. In those short-term exercise studies no significant gains in HRQoL were observed [22,27]. Because short-term training have been shown to produce only temporary improvements in various outcome measures, intensive resistance training for at least one year is recommended to gain sustainable results [28]. Thus, the purpose of the present study was to evaluate whether 12 months of neck strength or endurance training could improve HRQoL in females with chronic neck pain. This study was a secondary analysis of the randomized, controlled study conducted by Ylinen et al. [23].

## Methods

### Subjects

Three hundred forty-seven female office workers from different workplaces in southern and eastern Finland were referred to the study through their occupational health care systems. Potential subjects were identified through the local offices of the Social Insurance Institution, which provides state-financed rehabilitation in Finland. A questionnaire was mailed to these prospective participants to confirm their status regarding the inclusion and exclusion criteria. At this stage 121 candidates were excluded because of not meeting the eligibility criteria. Finally a total of 180 females met the inclusion criteria and also entered the study. Inclusion criteria were: female, aged 25 to 53 years, office worker, permanently employed, motivated to continue working, motivated for rehabilitation, and constant or frequently occurring neck pain for more than 6 months. Exclusion criteria were severe disorders of the cervical spine, such as disk prolapse, spinal stenosis, postoperative conditions in the neck and shoulder areas, history of severe trauma, instability, spasmodic torticollis, frequent migraine, peripheral nerve entrapment, fibromyalgia, shoulder diseases (tendonitis, bursitis, capsulitis), inflammatory rheumatic diseases, severe psychiatric illness and other diseases that prevent physical loading, and pregnancy. A detailed flowchart depicting the step-by-step enrolment process was published in an earlier report [23]. The subjects were randomized into two training groups and into a control group. A randomization into three groups of ten persons was performed blind before inviting the subjects to the rehabilitation centre. After obtaining 30 subjects, 10 in each group, they were ranked by the neck and shoulder pain and disability index and divided into 10 blocks of three groups. From each block, one subject was randomized to one of the training groups or to the control group according to a computer generated list. This stratification was used to ensure that subjects with equal severity of neck symptoms were present in each group. The trial was conducted between February 2000 and March 2002.

All of the participants provided written informed consent before entering the study. The study design was approved by the ethics committee of the Punkaharju Rehabilitation Centre, Punkaharju, Finland.

### Measurements

All measurements were performed blind by the same physical therapist at baseline and after the 12-month intervention period. HRQoL was measured using the generic self-administered questionnaire 15D, which includes the dimensions mobility, vision, hearing, breathing, sleeping, eating, speech, elimination, usual activities, mental function, discomfort and symptoms, depression, distress, vitality, and sexual activity [6]. Each dimension has five grades of severity. The 15D can be used both to obtain a profile across the 15 dimensions and a single index score ranging from 0 (being dead) to 1 (full health). The 15D has proven to be reliable and valid instrument for measuring HRQoL [6,29-31]. It has also been used to describe the impact of different chronic conditions on HRQoL, including neck problems [12].

A neck strength measurement system (Kuntoväline Ltd, Helsinki, Finland) was used to test the isometric neck muscle strength with patients seated in a standard position, and the methodology followed the same method used in the reliability study reported earlier [32].

### Interventions

The subjects were randomized into three groups: a strength training group (STG, *n *= 60), an endurance training group (ETG, *n *= 60), and a control group (CG, *n *= 60). Both of the training groups participated in a 12-day rehabilitation program in a rehabilitation centre; the program was then performed as a home training program for one year.

The STG used a rubber band to train the neck muscles in a single series of 15 repetitions, each repetition reaching resistance level of 80% of the patient's maximum isometric strength as recorded at baseline. The patient sat in an upright position and the other end of the rubber band was attached to the patients head and the other end to a sturdy stand. The patient then bent from hips directly forwards, obliquely toward right and left and directly backwards. The erect posture of the spine was maintained throughout the exercise. The subject's ability to reach the 80% resistance level was checked with a handheld isometric strength testing device (Force-Five, Wagner Instruments, Greenwich, CT) attached to the rubber band, at the baseline and at 2- and 6-month follow-up visits for controlling the progress of the training. In addition, a single adjustable dumbbell was used to perform upper body exercises: dumbbell shrugs, presses, curls, bent-over rows, flies, and pullovers. For each exercise, one set of 15 repetitions at the highest load possible was performed. Training was progressive such that if a patient could do 20 or more repetitions, weight was added.

The ETG trained their neck muscles by lifting the head up from supine position in three sets of 20 repetitions. The patients used a pair of dumbbells each weighing 2 kg to perform three sets of 20 repetitions of the same upper body exercises the STG was performing. Both training groups exercised three times per week and also performed a single series of squats, sit-ups, and back extension exercises in addition to 20 minutes of stretching exercises for the muscles trained.

The CG received written information and a single guidance session concerning the same stretching exercises that the training groups were performing. In addition, all the three groups were encouraged to perform aerobic exercise three times a week for 30 minutes.

Compliance with the specific training programs was collected via a training diary throughout the 12-month intervention. The training diaries were checked at 2-, 6-, and 12-month visits for the two training groups and at 12-month for the control group.

### Data analysis

The results are expressed as means and standard deviations (SD). Statistical comparisons between the groups in baseline characteristics were performed using analysis of variance. The differences between groups in 15D dimensions and total score were tested by using bootstrap techniques due to the skewed distributions. Bootstrapping is a re-sampling method, in which you make no assumptions on distribution [33]. A bootstrap-type analysis of variance was used to test differences at baseline. Changes between the groups were tested by bootstrap-type analysis of covariance (ANCOVA) with baseline values as covariates. Effect sizes were calculated using the Cohen method for paired samples [34]. An effect size of 0.20 was considered as small, 0.50 as medium, and 0.80 as large. Confidence intervals (95% CIs) for the effect sizes were obtained by bias-corrected bootstrapping (5,000 replications) [35]. Post hoc (observed) power calculation was done based on Monte Carlo simulation of ANOVA designs. The α-level was set at 0.05. All statistical analyses were performed using STATA (for Windows), version 10 (Stata Corp, College Station, TX, USA).

## Results

The mean (SD) age of the patients was 46 (6) years and the mean duration of neck pain was 8 (6) years. The demographic and clinical characteristics of the study groups were similar at baseline (table 1).

**Table 1:** Characteristics of the study participants

Variable	Training groups			P value‡
		
	Control group *n *= 60 Mean (SD)	Endurance *n *= 59 Mean (SD)	Strength *n *= 60 Mean (SD)	
Demographic				
Age, years	46 (5)	46 (6)	45 (6)	0.73
Height, cm	164 (5)	165 (6)	165 (5)	0.74
Weight, kg	69 (12)	68 (10)	67 (11)	0.64
Body mass index	26 (4)	25 (3)	25 (3)	0.40
Clinical characteristic				
Duration of neck pain, years	8 (5)	9 (6)	8 (6)	0.30
Neck pain, mm (VAS†, scale 0-100)	58 (20)	56 (22)	57 (20)	0.89

†Visual Analog Scale

‡ P value with ANOVA

One patient in the endurance training group was excluded after randomization because of diagnosed polymyalgia rheumatica. Another patient withdrew from the endurance training group because of personal reason and one patient withdrew from the control group due to pregnancy. There were no missing data in addition to the two drop-outs.

At 12 months, changes in the 15D total scores (P = 0.012; observed power 0.76, α = 0.05) and the dimension sleeping (P = 0.0019) between the groups were statistically significant (Additional file 1, Table S2). Statistically significant gains in the 15D total score were observed for both training groups, whereas no changes occurred for the CG. There were statistically significant gains in the dimensions sleeping, elimination, mental function, distress, and vitality in the STG and in the dimensions sleeping and vitality in the ETG. In the CG, statistically significant deterioration was observed in the dimension mental function.

**Figure 1:**
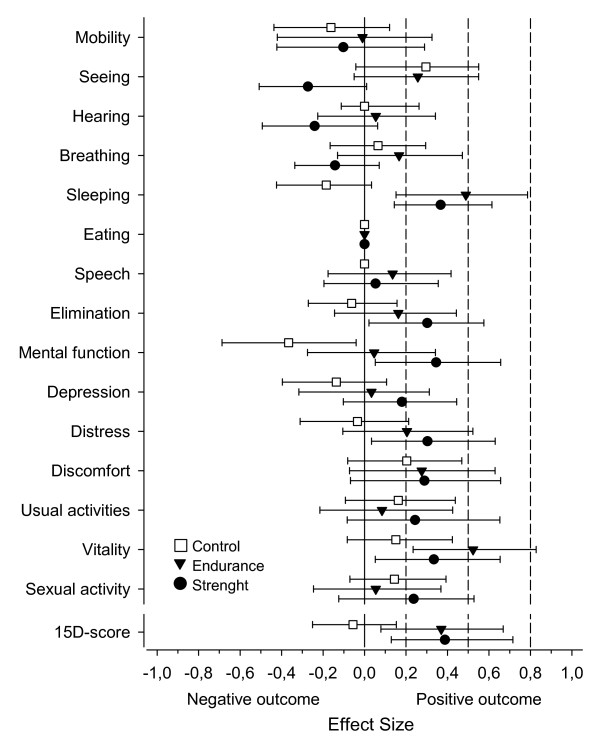
**Effect sizes of the 15 dimensions and total score of the 15D**. Error bars indicate 95% confidence intervals. Small (0.20), medium (0.50), and large (0.80) effect sizes are illustrated with dotted lines.

Effect size (95% CI) for the 15D total score was 0.39 (0.13 to 0.72) for the STG, 0.37 (0.08 to 0.67) for the ETG, and -0.06 (-0.25 to 0.15) for the CG. A medium-sized positive effect was observed in the ETG for the dimension vitality (mean, 0.52; 95% CI, 0.23 to 0.83; Figure 1).

## Discussion

This study showed that twelve months of neck strength or endurance training significantly improved HRQoL compared to control group among females with chronic neck pain. Both training groups showed statistically significant improvements in the 15D total score. The STG improved significantly in five of 15 dimensions, whereas the ETG improved in two of 15 dimensions.

The effect sizes for the 15D and its subscales in the present study seem to be modest. Nevertheless, Dr. Sintonen the developer of the 15D has stated that a change of 0.02 to 0.03 is clinically relevant for people in the sense that they feel the difference [36]. Since the statistically significant improvements in 15D and its dimensions ranged from 0.024 to 0.059 in the STG and from 0.021 to 0.068 in the ETG, it can be suggested that these improvements were also clinically relevant. Especially so, as such improvement was not observed in the control group.

HRQoL measurements have seldom been reported as outcomes in exercise intervention studies exploring chronic neck pain. The SF-36 HRQoL measurement was applied in two short-term intervention studies. Bronfort et al. [22] compared the effects of spinal manipulation combined with neck exercises, rehabilitative neck exercises alone, and spinal manipulation alone on neck pain. After 11 weeks of intervention, minor improvements were observed among all groups in all outcome measures including SF-36, but they did not reach statistical significance. Helewa et al. [27] investigated the effects of therapeutic exercises and sleeping with neck support pillows in patients with neck pain. The patients were treated for 6 weeks and the primary assessment was performed at 12 weeks. No statistically significant differences in HRQoL were detected among the groups.

There are some differences between the studies of Bronfort et al. [22] and Helewa et al. [27] and the present study. The most conspicuous of these is the length of the intervention, which was 12 months in the present study and less than 3 months in the aforementioned studies. According to Ylinen [28], the length of the commitment to regular training is one of the key factors for lasting rehabilitation results for chronic neck pain. Only a few months of training have been shown to produce only temporary improvements in various outcome measures; thus, intensive resistance training for at least one year is recommended [28]. In the original study by Ylinen et al. [23] the 12 month training led to statistically significant pain reduction in the STG and ETG compared to the CG. While neck pain is shown to be associated with a decrease in HRQoL in earlier cross-sectional studies [1,7-12] the present reduction in pain may be one factor responsible for the significant enhancement in HRQoL in the STG and ETG compared to the CG. In addition to the long training period, compliance to the training method used was good. The training adherence (at least once a week) was 86% for the STG, 93% for the ETG, and 65% for the CG [37]. Time used to aerobic exercise did not differ between groups at baseline or at 12-months. Also, no other treatments were offered to the patients during the 12-month period and visits to a physician and use of therapies e.g. massage was decreased especially in the STG and ETG during the 12 month period. The use of other treatments is described in details in the original report by Ylinen et al. [23].

There seems to be also some limitations in the study. While there were differences in HRQoL at baseline among groups, regression to the mean might explain some of the changes at 12 months. For example mental function scores were significantly higher at baseline in the CG compared to STG and ETG, and deterioration of mental function in CG at 12 months might be hard to explain otherwise than by tendency of abnormal values to average towards the mean of the population. By including a group of healthy volunteers to explore how much the 15D values fluctuate during one year, the conclusions of the present study could have been strengthened. The study group was selected through a long selection procedure which is possible to have influenced leaving out the least motivated patients. This might explain the high compliance and good completion of questionnaires so that there was no missing data except the two cases that withdrew from the study. Results in other settings e.g. in outpatient clinics, might differ from the present findings. Thus further studies are needed in other settings and especially among men.

## Conclusions

One year of either strength or endurance training seemed to moderately enhance the HRQoL of female patients with chronic neck pain. Neck and upper body training can be recommended to improve HRQoL of females with neck pain if they are motivated for long-term regular exercise.

## Competing interests

The authors declare that they have no competing interests.

## Authors' contributions

PS was involved in the statistical analysis and drafted the manuscript. AH participated in the statistical analysis and drafting of the manuscript. HK performed the statistical analysis and participated in drafting of the manuscript. JY was the principal investigators of the original study and prepared study design, data collection and participated in drafting of the manuscript. All authors read and approved the final manuscript.

## Supplementary Material

Additional file 1 **Table S2: HQoLO**. 15D ratings of groups at baseline and after 12 months. Click here for file
