# Supplementary material for: Effect of neck strength training on health-related quality of life in females with chronic neck pain: a randomized controlled 1-year follow-up study
Source: Health Qual Life Outcomes. 2010 May 14;8:48. doi: 10.1186/1477-7525-8-48 (PMC2877013; doi:10.1186/1477-7525-8-48)
Supplement: Additional file 1 — Table S2: HQoLO. 15D ratings of groups at baseline and after 12 months. [file 1477-7525-8-48-S1.DOC]

|  | Baseline  Mean (SD) | | |  | Change after 12 months  Mean (95% CI) | | |  | *P*-value between groups* |
| --- | --- | --- | --- | --- | --- | --- | --- | --- | --- |
|  | Controls  CG  *n* = 60 | Endurance  ETG  *n* = 59 | Strength  STG  *n* = 60 |  | Controls  *n* = 59 | Endurance  *n* = 58 | Strength  *n* = 60 |  |  |
| Dimensions |  |  |  |  |  |  |  |  |  |
| Mobility | 0.971 (0.087) | 0.958 (0.128) | 0.981 (0.072) |  | -0.015 (-0.040 to 0.011) | -0.001 (-0.044 to 0.042) | -0.009 (-0.041 to 0.023) |  | 0.82 |
| Seeing | 0.944 (0.131) | 0.960 (0.085) | 0.978 (0.065) |  | 0.031 (-0.005 to 0.068) | 0.019 (-0.003 to 0.040) | -0.026 (-0.055 to 0.004) |  | 0.16 |
| Hearing | 0.983 (0.063) | 0.966 (0.086) | 0.988 (0.055) |  | 0.000 (-0.012 to 0.012) | 0.004 (-0.018 to 0.027) | -0.017 (-0.037 to 0.003) |  | 0.41 |
| Breathing | 0.921 (0.141) | 0.904 (0.178) | 0.965 (0.098) |  | 0.009 (-0.022 to 0.040) | 0.026 (-0.024 to 0.076) | -0.015 (-0.037 to 0.007) |  | 0.91 |
| Sleeping | 0.809 (0.132) | 0.761 (0.153) | 0.793 (0.142) |  | -0.025 (-0.056 to 0.006) | 0.066 (0.025 to 0.107) | 0.052 (0.018 to 0.085) |  | 0.0019 † |
| Eating | 1.000 (0.000) | 1.000 (0.000) | 0.994 (0.046) |  | 0.000 (0.000 to 0.000) | 0.000 (0.000 to 0.000) | 0.000 (0.000 to 0.000) |  |  |
| Speech | 0.980 (0.075) | 0.970 (0.090) | 0.965 (0.096) |  | 0.000 (0.000 to 0.000) | 0.010 (-0.010 to 0.030) | 0.005 (-0.020 to 0.030) |  | 0.65 |
| Elimination | 0.911 (0.143) | 0.878 (0.173) | 0.881 (0.189) |  | -0.010 (-0.043 to 0.023) | 0.027 (-0.024 to 0.078) | 0.052 (0.002 to 0.101) |  | 0.23 |
| Mental function | 0.947 (0.128) | 0.891 (0.166) | 0.859 (0.184) |  | -0.054 (-0.102 to -0.007) | 0.008 (-0.041 to 0.057) | 0.059 (0.009 to 0.110) |  | 0.16 |
| Depression | 0.922 (0.112) | 0.904 (0.133) | 0.886 (0.142) |  | -0.016 (-0.047 to 0.015) | 0.004 (-0.036 to 0.045) | 0.024 (-0.012 to 0.060) |  | 0.67 |
| Distress | 0.890 (0.136) | 0.889 (0.144) | 0.854 (0.153) |  | -0.005 (-0.043 to 0.034) | 0.028 (-0.016 to 0.072) | 0.046 (0.004 to 0.088) |  | 0.41 |
| Discomfort | 0.693 (0.150) | 0.698 (0.146) | 0.727 (0.139) |  | 0.031 (-0.008 to 0.070) | 0.044 (-0.010 to 0.098) | 0.045 (-0.008 to 0.098) |  | 0.46 |
| Usual activities | 0.930 (0.122) | 0.934 (0.120) | 0.921 (0.127) |  | 0.019 (-0.010 to 0.048) | 0.010 (-0.028 to 0.048) | 0.033 (-0.012 to 0.078) |  | 0.90 |
| Vitality | 0.820 (0.123) | 0.796 (0.134) | 0.835 (0.143) |  | 0.019 (-0.012 to 0.051) | 0.068 (0.032 to 0.105) | 0.046 (0.006 to 0.086) |  | 0.15 |
| Sexual activity | 0.952 (0.109) | 0.961 (0.100) | 0.933 (0.133) |  | 0.015 (-0.011 to 0.040) | 0.005 (-0.021 to 0.031) | 0.029 (-0.010 to 0.068) |  | 0.99 |
| Total 15D score | 0.912 (0.050) | 0.896 (0.063) | 0.903 (0.059) |  | -0.003 (-0.013 to 0.007) | 0.021 (0.005 to 0.037) | 0.024 (0.009 to 0.039) |  | 0.012 ‡ |

*Overall statistical significance between groups for change after 12 months; bootstrap (5,000 replicas)-type ANCOVA with baseline values as covariates: post hoc comparison † (ETG vs. CG and STG vs. CG) ‡ (ETG vs. CG and STG vs. CG).

Overall statistical significance between groups at baseline in dimensions Breathing (p=0.025) (CG vs. STG, ETG vs. STG) and Mental function (p=0.0057) (CG vs. ETG and CG vs. STG).
